# Supplementary material for: Frontotemporal dementia-like disease progression elicited by seeded aggregation and spread of FUS
Source: Mol Neurodegener. 2024 Jun 11;19:46. doi: 10.1186/s13024-024-00737-5 (PMC11165889; doi:10.1186/s13024-024-00737-5)
Supplement: Supplementary file 1 — Supplementary Material 1. [file 13024_2024_737_MOESM1_ESM.pdf]

Additional File 1:

Frontotemporal dementia-like disease progression from age-dependent, seeded aggregation and spread of FUS

Sonia Vazquez-Sanchez[1#], Britt Tilkin[2#], Fatima Gasset-Rosa[1#^], Sitao Zhang[1], Diana Piol[2], Melissa McAlonis-Downes[1], Jonathan Artates[1], Noe Govea-Perez[1], Yana Verresen[2], Lin Guo[3], James Shorter[4], Don W. Cleveland[1], and Sandrine Da Cruz[2*]

Affiliations

1. Department of Cellular and Molecular Medicine, University of California at San Diego, La Jolla, CA 92093, USA
2. VIB-KU Leuven Center for Brain and Disease Research and Department of Neurosciences, KU Leuven, Leuven 3000, Belgium
3. Thomas Jefferson University, Philadelphia, PA 19107, USA
4. Department of Biochemistry & Biophysics, University of Pennsylvania, Philadelphia, PA 19104-6059, USA

[#] These authors contributed equally to the work.

^Present address: Vividion Therapeutics, 5820 Nancy Ridge Dr, San Diego, CA 92121

*Corresponding author: Sandrine Da Cruz, sandrine.dacruz@kuleuven.be

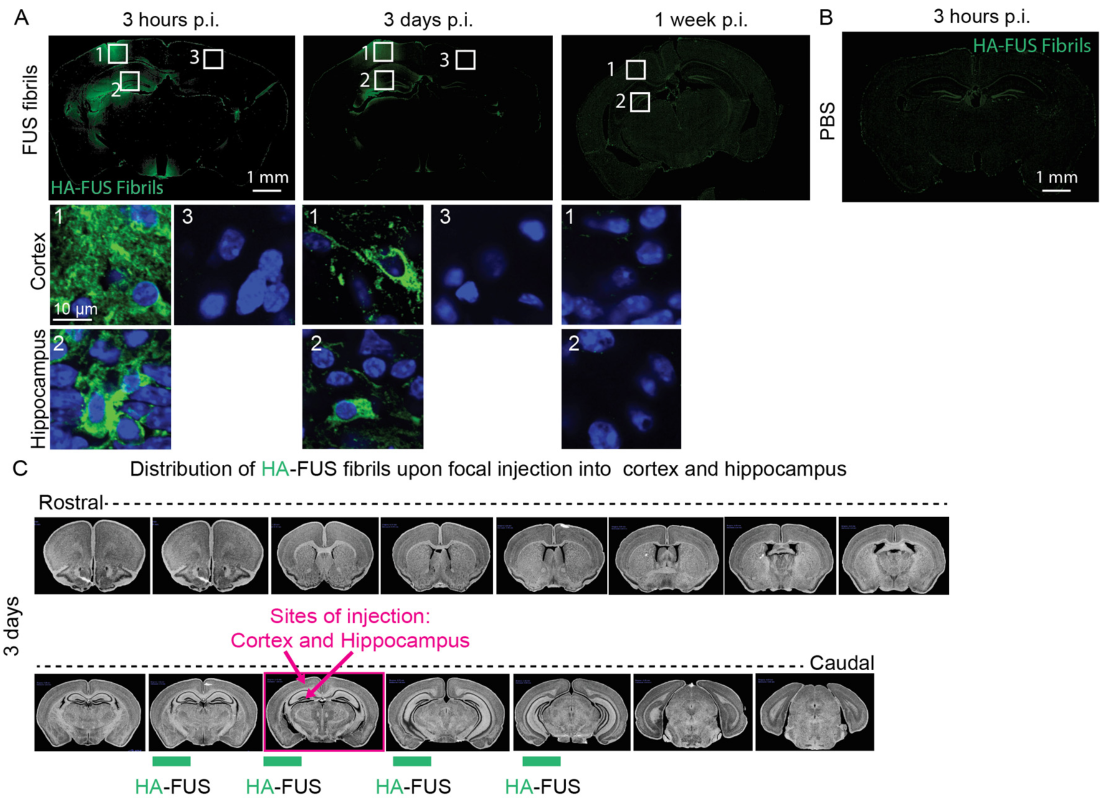

Figure S1: Distribution of focally-injected HA-tagged FUS^{R495X} fibrils in the mouse brain at 3 hours, 3 days and 1-week post-injection

A. Immunostaining of cortex and hippocampus of mFUS^{KO}/hFUS^{R521H} mouse brain sections 3 hours, 3 days and 1-week post-injection with HA-FUS^{R495X} fibrils using an anti-HA antibody to detect the HA-tagged FUS^{R495X} fibrils. Number 1-2 correspond to the side of injection, number 3 corresponds with the contralateral side. DAPI as nuclear counterstaining. Scale bar: 10 µm. **B.** Immunostaining of mFUS^{KO}/hFUS^{R521H} mouse brain sections 3 hours post-injection with PBS using an anti-HA antibody. **C.** Illustration summarizing the distribution of HA-FUS^{R495X} fibrils throughout the 3-day post-injection. Red dots indicate the sites where FUS aggregates are immunodetected.

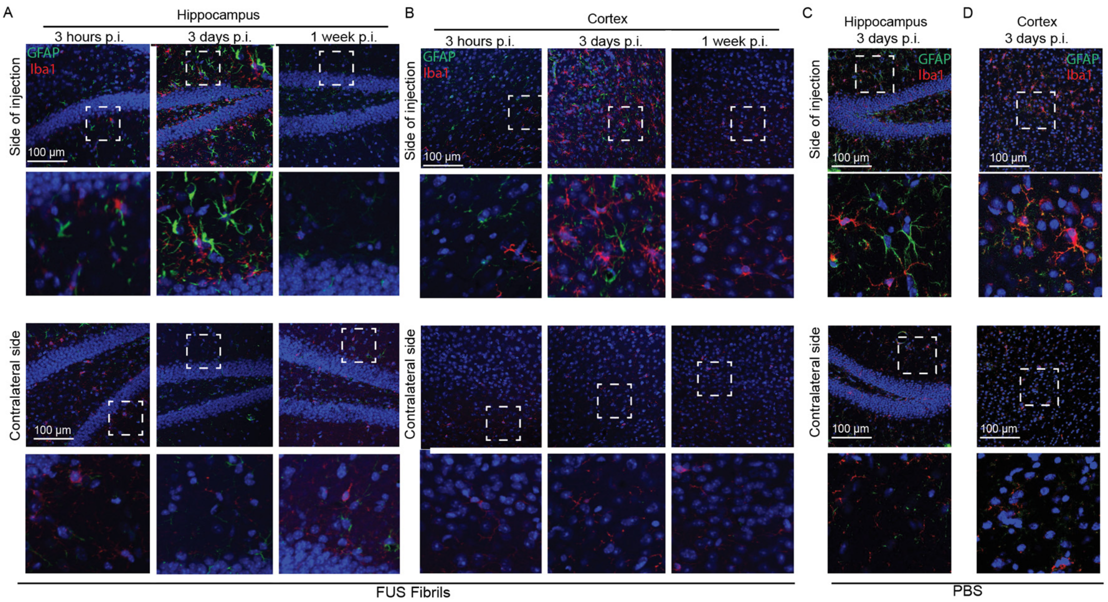

Figure S2: Focal stereotactic injections induce transient activation of glial cells
Representative confocal images of the site of injection in hippocampus (**A**) and cortex (**B**) of mFUS^{KO}/hFUS^{R521H} mice injected with HA-FUS^{R495X} fibrils, 3 hours, 3 days or 1-week post-injection immunolabeled for GFAP (astrocytes) and Iba1 (microglia). DAPI as nuclear counterstaining. Representative confocal images of the site of injection in hippocampus (**C**) and cortex (**D**) of mFUS^{KO}/hFUS^{R521H} mice injected with PBS, 3 days post-injection immunolabeled for GFAP (astrocytes) and Iba1 (microglia). DAPI is used as nuclear counterstaining. Scale bar: 100 µm.

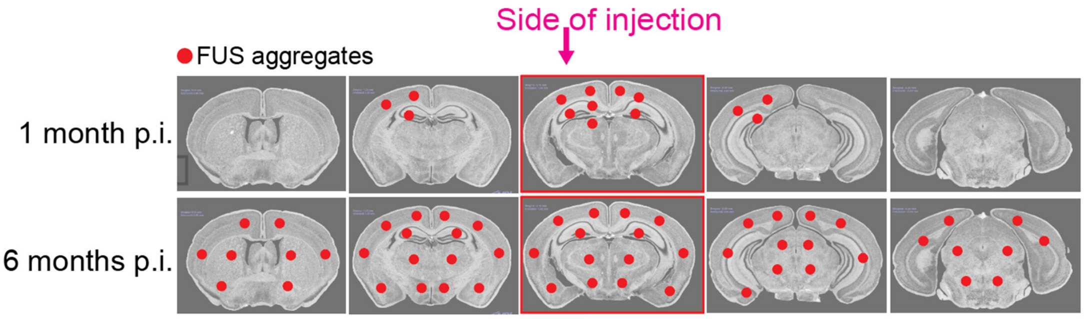

Figure S3: Distribution of FUS cytoplasmic aggregates in mFUS^{KO}/hFUS^{R521H} mice induced by HA-FUS^{R495X} fibrils

Illustration summarizing the distribution of FUS cytoplasmic aggregates throughout the brain, 1- and 6-months post-injection. Red dots indicate the sites where FUS aggregates are immunodetected.

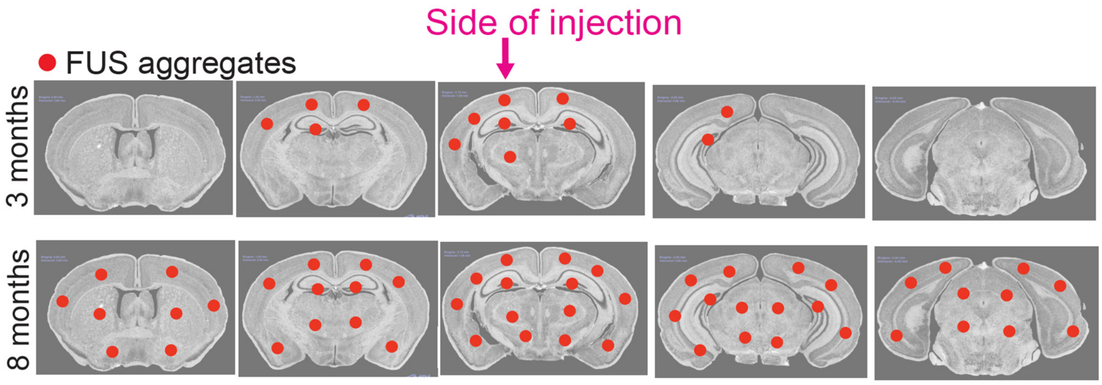

Figure S4: Distribution of FUS cytoplasmic aggregates in mFUS^{KO}/hFUS^{WT} mice induced by HA-FUS^{R495X} fibrils

Illustration summarizing the distribution of FUS cytoplasmic aggregates throughout the brain, 3- and 8-months post-injection. Red dots indicate the sites where FUS aggregates are immunodetected.

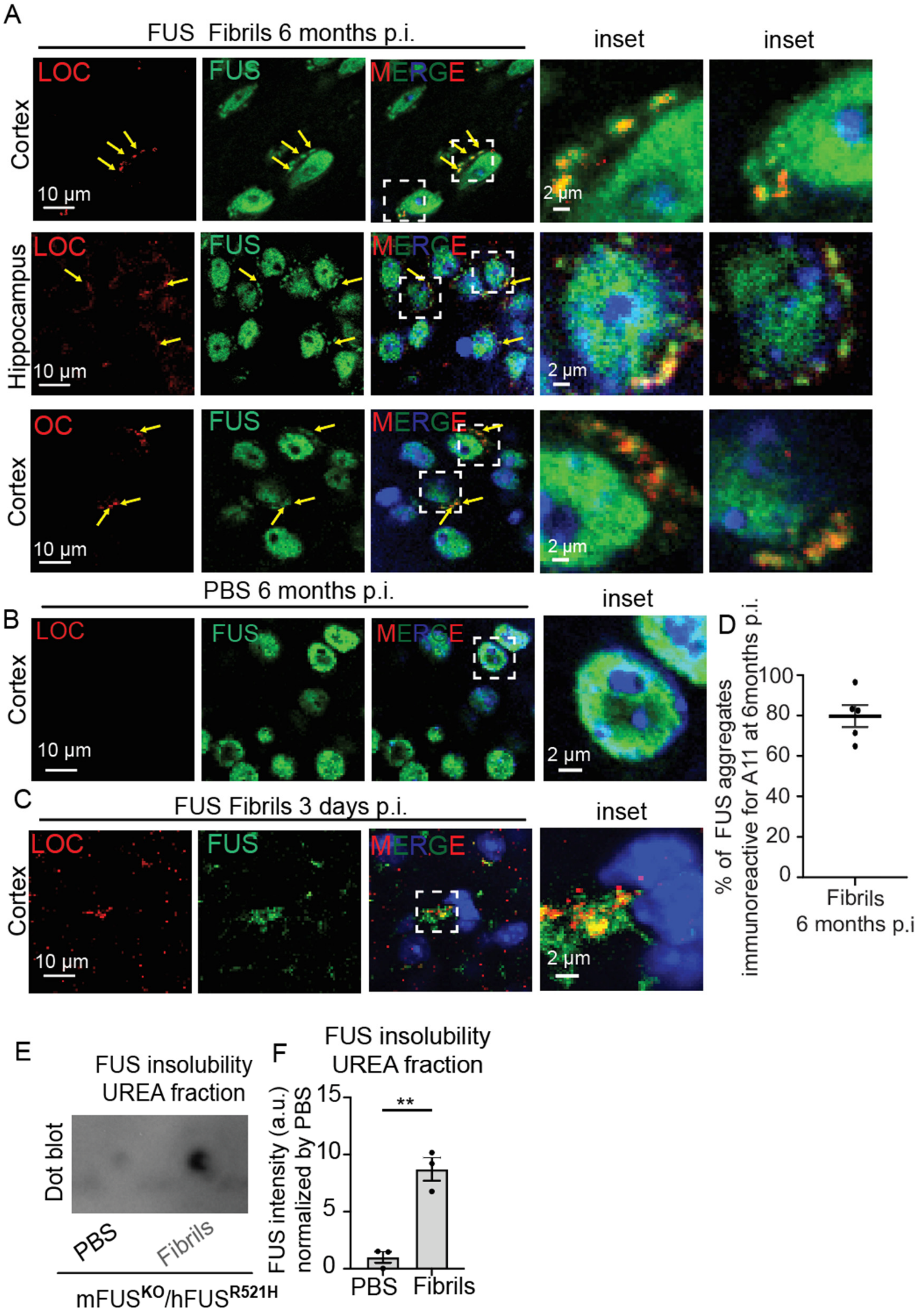
A
FUS Fibrils 6 months p.i.
inset
inset
Cortex
LOC
FUS
MERGE
10 µm
2 µm
Hippocampus
LOC
FUS
MERGE
10 µm
2 µm
Cortex
OC
FUS
MERGE
10 µm
2 µm
PBS 6 months p.i.
inset
B
Cortex
LOC
FUS
MERGE
10 µm
2 µm
C
FUS Fibrils 3 days p.i.
inset
Cortex
LOC
FUS
MERGE
10 µm
2 µm
D
% of FUS aggregates immunoreactive for A11 at 6months p.i.
100
80
60
40
20
0
Fibrils
6 months p.i
E
FUS insolubility
UREA fraction
Dot blot
PBS
Fibrils
mFUSKO/hFUSR521H
F
FUS insolubility
UREA fraction
FUS intensity (a.u.) normalized by PBS
15
10
5
0
**
PBS
Fibrils

Figure S5: FUS aggregates display amyloid properties

A,B. Representative confocal micrographs of $mFUS^{KO}/hFUS^{R521H}$ mouse brains injected with $HA\text{-}FUS^{R495X}$ fibrils, at 6 months post-injection (panel A) immunolabelled with amyloid fibril markers LOC and OC (red) and FUS (green). Yellow arrows indicate co-localization between LOC/OC and FUS cytoplasmic inclusions in fibril-injected mice but not in PBS-injected controls (panel B). **C**. Co-localization between LOC (red) and $HA\text{-}FUS^{R495X}$ fibrils (green) 3 days post-injection. DAPI (blue) as nuclear counterstaining. Scale bars: 10 µm, inset: 2 µm. **D**. Quantification of the percentage of FUS aggregates that are A11-positive in $HA\text{-}FUS^{R495X}$ fibril injected $mFUS^{KO}/hFUS^{R521H}$ mice 6 months post-injection. N=3 animals. **E**. Dot-blot analysis of FUS protein levels in urea insoluble fractions of PBS and $HA\text{-}FUS^{R495X}$ fibril injected $mFUS^{KO}/hFUS^{R521H}$ mouse homogenates using FUS antibody. **F**. Quantification of FUS protein levels in urea insoluble fractions shown in E.

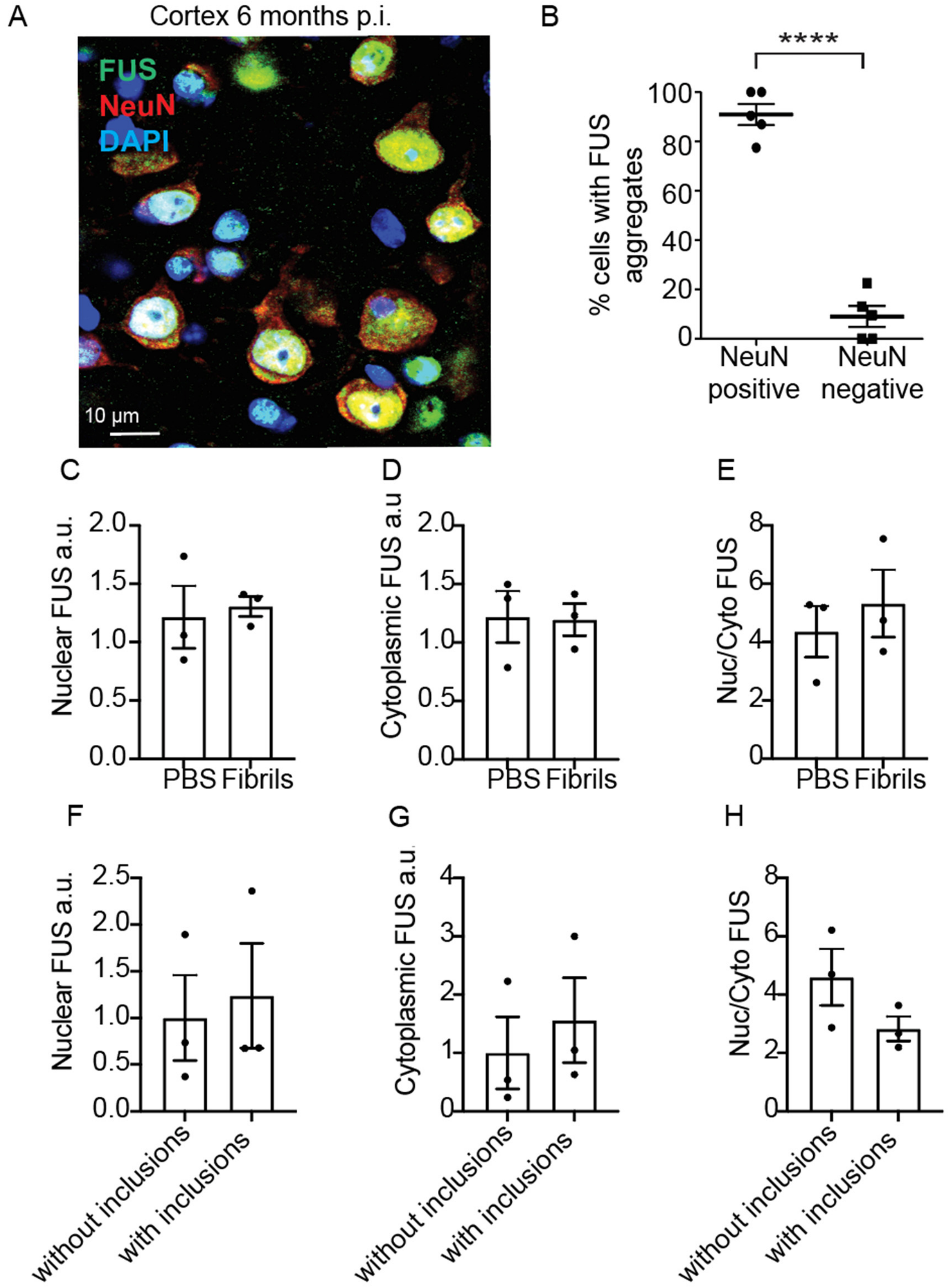
A
Cortex 6 months p.i.
FUS
NeuN
DAPI
10 µm
B
****
% cells with FUS aggregates
100
80
60
40
20
0
NeuN positive
NeuN negative
C
Nuclear FUS a.u.
2.0
1.5
1.0
0.5
0.0
PBS
Fibrils
D
Cytoplasmic FUS a.u
2.0
1.5
1.0
0.5
0.0
PBS
Fibrils
E
Nuc/Cyto FUS
8
6
4
2
0
PBS
Fibrils
F
Nuclear FUS a.u.
2.5
2.0
1.5
1.0
0.5
0.0
without inclusions
with inclusions
G
Cytoplasmic FUS a.u.
4
3
2
1
0
without inclusions
with inclusions
H
Nuc/Cyto FUS
8
6
4
2
0
without inclusions
with inclusions

Figure S6: FUS aggregation accumulates predominantly in neurons of fibril-injected $mFUS^{KO}/hFUS^{R521H}$ mice

A. Immunostaining of $mFUS^{KO}/hFUS^{R521H}$ mouse brains injected with HA-FUS^{R495X} fibrils for neuronal marker NeuN (red) and FUS (green) 6 months post-injection. Scale bars: 10 µm. **B.** Quantification of the percentage of NeuN-positive cells and NeuN-negative cells that contain FUS cytoplasmic aggregation. DAPI as nuclear counterstaining. N = 5. **C.** Quantification of the FUS nuclear intensity signal in neurons of both ipsi- and contra-lateral side mice injected either with PBS or with HA-FUS^{R495X} fibrils 6 months post-injection. N = 3 mice per group. **D.** Quantification of the FUS cytoplasmic intensity signal in neurons of both ipsi- and contra-lateral side mice injected either with PBS or with HA-FUS^{R495X} fibrils 6 months post-injection. N = 3 mice per group. **E.** Quantification of the FUS nuclear/cytoplasmic ratio in neurons of both ipsi- and contra-lateral side mice injected either with PBS or with HA-FUS^{R495X} fibrils 6 months post-injection. N = 3 mice per group. **F.** Quantification of nuclear FUS, **G.** cytoplasmic FUS and **H.** FUS nuclear/cytoplasmic ratio in neurons with and without FUS cytoplasmic inclusions of mice injected with HA-FUS^{R495X} fibrils 6 months post-injection. N = 3 mice.

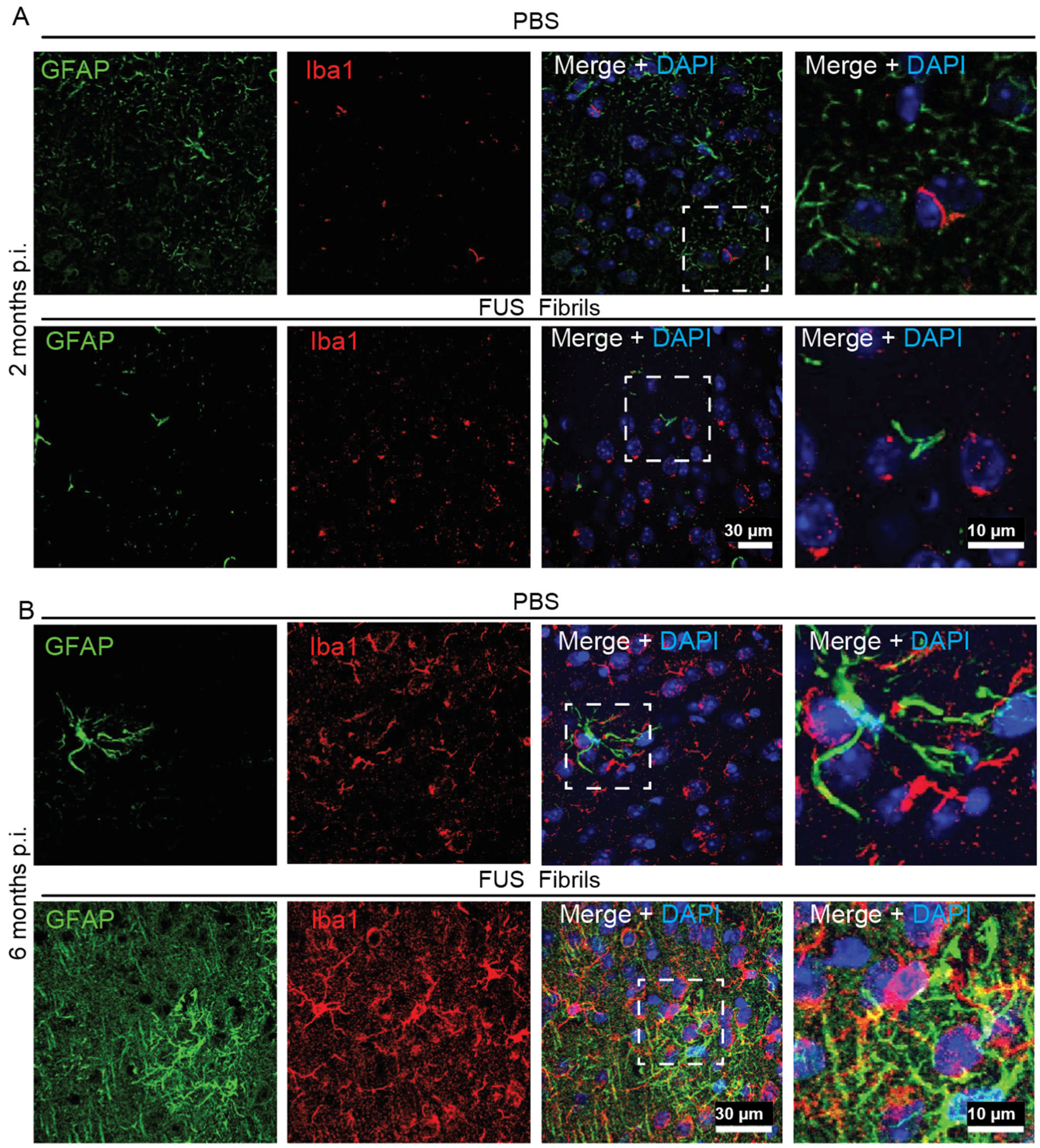

Figure S7: FUS aggregation is accompanied by increased gliosis in fibril-injected mFUS^{KO}/hFUS^{R521H} mice

Immunostaining for astrocyte marker GFAP (green) and microglial marker Iba1 (red) of cortex of mFUS^{KO}/hFUS^{R521H} mice at 2 (**A**) and 6 months (**B**) post-injection either with PBS or with HA-FUS^{R495X} fibrils. Scale bars: 30 and 10 µm.

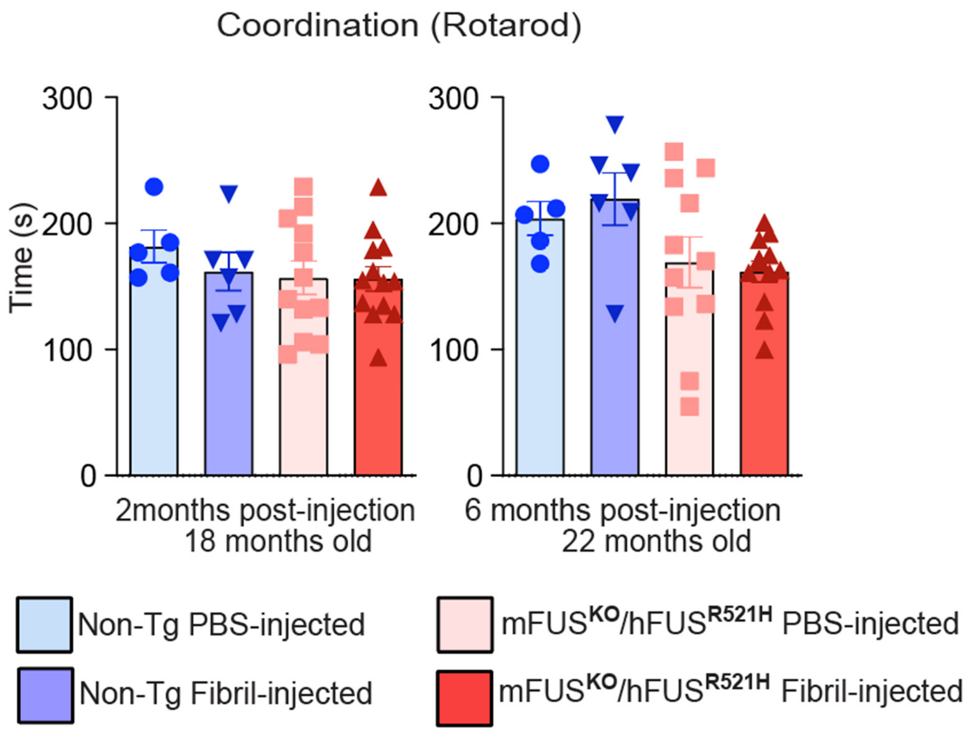

Figure S8: Rotarod performance in non-transgenic and humanized mutant FUS mice injected either with PBS or sonicated FUS fibrils

Rotarod test was performed in 22 months old HA-FUS^{R495X} fibril injected mFUS^{KO}/hFUS^{R521H} animals (2 and 6 months post-injection) compared to PBS-injected controls and non-transgenic HA-FUS^{R495X} fibrils or PBS injected controls. N=5–12 animals per group. Data is presented as mean ± SEM.

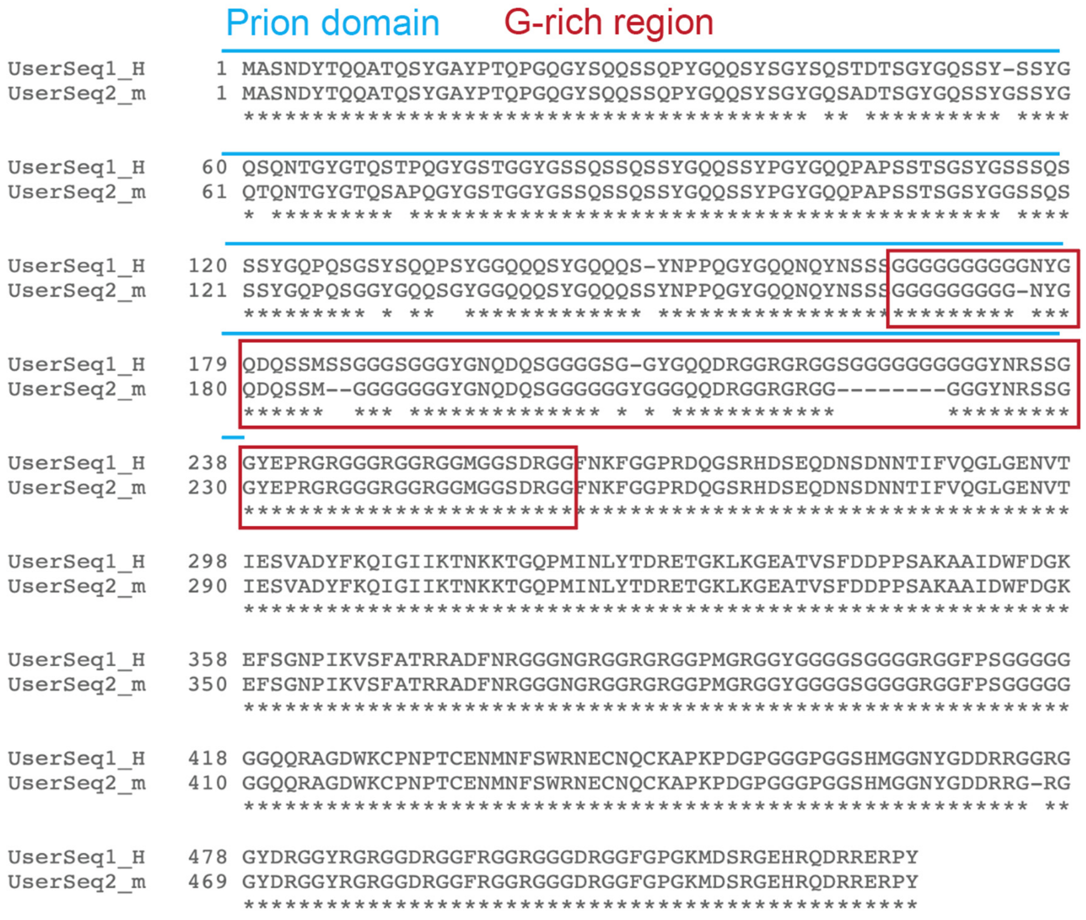

Figure S9: Comparison of human and mouse FUS protein sequences

Supplementary Tables:

Supplementary Table S1: Statistical summary corresponding to Figure 1

Figure	Mouse genotype	Fibrils injected	Measured variable	Brain region	Kruskal-Wallis test	Dunn's multiple comparisons test	p-value
1F	$mFUS^{KO}/hFUS^{R521H}$	HA-FUS^{R495X} fibrils	%of cells with cytoplasmic aggregates	Cortex ipsilateral	p = 0.0203	1 month vs. 2 months	0.4116
						1 month vs. 6 months	0.0429
						2 months vs. 6 months	>0.9999
				Hippocampus ipsilateral	p = 0.0036	1 month vs. 2 months	0.5391
						1 month vs. 6 months	0.0219
						2 months vs. 6 months	0.5391
1I	$mFUS^{KO}/hFUS^{R521H}$	HA-FUS^{R495X} fibrils	%of cells with cytoplasmic aggregates	Cortex contralateral	p = 0.0750	1 month vs. 2 months	0.5934
						1 month vs. 6 months	0.0225
						2 months vs. 6 months	0.091
				Hippocampus contralateral	p = 0.0107	not compared	
						not compared	
						not compared	

Supplementary Table S2: Statistical summary corresponding to Figure 2

Figure	Mouse genotype	Fibrils injected	Measured variable	Brain region	Kruskal-Wallis test	Dunn's multiple comparisons test	p-value
2I	$mFUS^{KO}/hFUS^{R521H}$	HA-FUS^{R495X} fibrils	%of cells with cytoplasmic aggregates	Cortex ipsilateral	p = 0.0036	1 month vs. 3 months	0.5172
						1 month vs. 8 months	0.019
						3 months vs. 8 months	0.5172
				Cortex contralateral	p = 0.0071	1 month vs. 3 months	0.7558
						1 month vs. 8 months	0.0312
						3 months vs. 8 months	0.7558
				Hippocampus ipsilateral	p = 0.0071	1 month vs. 3 months	0.3884
						1 month vs. 8 months	0.0299
						3 months vs. 8 months	0.8656
				Hippocampus contralateral	p = 0.0036	1 month vs. 3 months	0.5172
						1 month vs. 8 months	0.019
						3 months vs. 8 months	0.5172

Supplementary Table S3: Statistical summary corresponding to Figure 4A and 4B

Figure	Measured variable	Mouse genotype	Material injected	Unpaired t-test p-value
4A	Recognition index	non-transgenic	PBS	0.7058
			HA-$FUSR^{495X\ fibrils}$	
		$mFUS^{KO}/hFUS^{R521H}$	PBS	0.0293
			HA-$FUSR^{495X\ fibrils}$	
4B	Time (s)	non-transgenic	PBS	0.8591
			HA-$FUSR^{495X\ fibrils}$	
		$mFUS^{KO}/hFUS^{R521H}$	PBS	0.0498
			HA-$FUSR^{495X\ fibrils}$	

Supplementary Table S4: Statistical summary corresponding to Figure 4D and 4E

Figure	Measured variable	Mouse genotype	Material injected	one-way ANOVA p-value	Tukey's multiple comparisons test	p-value
4D	Neurons per mm^2 in hippocampus	$mFUS^{KO}/hFUS^{R521H}$	Non	p = 0.0019	Non injected vs PBS	0.3281
			PBS		Non injected vs HA-$FUS^{R495X\ fibrils}$	0.0013
			HA-$FUSR^{495X\ fibrils}$		PBS vs HA-$FUS^{R495X\ fibrils}$	0.0631
4E	Neurons per mm^2 in cortex	$mFUS^{KO}/hFUS^{R521H}$	Non	p = 0.0026	Non injected vs PBS	0.315
			PBS		Non injected vs HA-$FUS^{R495X\ fibrils}$	0.0019
			HA-$FUSR^{495X\ fibrils}$		PBS vs HA-$FUS^{R495X\ fibrils}$	0.0574

Supplementary Table S5: Statistical summary corresponding to Supplementary S5

Figure	Measured variable	Mouse genotype	Material injected	Compared groups	Unpaired t-test p-value
S5F	FUS intensity (a.u.) in urea fraction	$mFUS^{KO}/hFUS^{R521H}$	HA-$FUSR^{495X\ fibrils}$	PBS	0.0024
				Fibril injected	

Supplementary Table S6: Statistical summary corresponding to Supplementary S6

Figure	Measured variable	Mouse genotype	Material injected	Compared groups	Unpaired t-test p-value
S6B	% of cells with FUS aggregates	$mFUS^{KO}/hFUS^{R521H}$	$HA\text{-}FUS^{R495X\ fibrils}$	NeuN -	<0.0001
				NeuN +	
S6C	FUS nuclear intensity (a.u.)	$mFUS^{KO}/hFUS^{R521H}$	$HA\text{-}FUS^{R495X\ fibrils}$	PBS	0.7603
				Fibril injected	
S6D	FUS cytoplasmic intensity (a.u.)	$mFUS^{KO}/hFUS^{R521H}$	$HA\text{-}FUS^{R495X\ fibrils}$	PBS	0.9303
				Fibril injected	
S6E	FUS nuclear/cytoplasmic ratio	$mFUS^{KO}/hFUS^{R521H}$	$HA\text{-}FUS^{R495X\ fibrils}$	PBS	0.5428
				Fibril injected	
S6F	FUS nuclear intensity (a.u.)	$mFUS^{KO}/hFUS^{R521H}$	$HA\text{-}FUS^{R495X\ fibrils}$	without inclusions	0.7592
				with inclusions	
S6G	FUS cytoplasmic intensity (a.u.)	$mFUS^{KO}/hFUS^{R521H}$	$HA\text{-}FUS^{R495X\ fibrils}$	without inclusions	0.5919
				with inclusions	
S6H	FUS nuclear/cytoplasmic ratio	$mFUS^{KO}/hFUS^{R521H}$	$HA\text{-}FUS^{R495X\ fibrils}$	without inclusions	0.1695
				with inclusions	

Supplementary Table S7: Statistical summary corresponding to Supplementary S8

Figure	Measured variable	Mouse genotype	Material injected	Unpaired t-test p-value
S8	Time (s)	non-transgenic	PBS	0.5656
			$HA\text{-}FUSR^{495X\ fibrils}$	
		$mFUS^{KO}/hFUS^{R521H}$	PBS	0.7252
			$HA\text{-}FUSR^{495X\ fibrils}$	
